# Predictors of Health-Related Quality of Life in Patients with Co-Morbid Diabetes and Chronic Kidney Disease

**DOI:** 10.1371/journal.pone.0168491

**Published:** 2016-12-19

**Authors:** Edward Zimbudzi, Clement Lo, Sanjeeva Ranasinha, Martin Gallagher, Gregory Fulcher, Peter G. Kerr, Grant Russell, Helena Teede, Tim Usherwood, Rowan Walker, Sophia Zoungas

**Affiliations:** 1 Department of Nephrology, Monash Health, Melbourne, Australia; 2 Monash Centre for Health Research and Implementation (MCHRI), School of Public Health and Preventive Medicine, Monash University, Melbourne, Australia; 3 Diabetes and Vascular Medicine Unit, Monash Health, Clayton, Melbourne, Australia; 4 Concord Clinical School, University of Sydney, Sydney, Australia; 5 The George Institute for Global Health, Sydney, Australia; 6 Department of Diabetes, Endocrinology & Metabolism, Royal North Shore Hospital, Sydney, Australia; 7 Northern Clinical School, University of Sydney, Royal North Shore Hospital, Sydney, Australia; 8 Southern Academic Primary Care Research Unit. School of Primary Health Care, Monash University, Melbourne, Australia; 9 The George Institute for Global Health, University of Sydney, Sydney, Australia; 10 Department of General Practice, Sydney Medical School Westmead, Sydney, Australia; 11 Department of Renal Medicine, the Alfred Hospital, Melbourne, Australia; Universidade Estadual Paulista Julio de Mesquita Filho, BRAZIL

## Abstract

**Background:**

People living with diabetes and chronic kidney disease (CKD) experience compromised quality of life. Consequently, it is critical to identify and understand factors influencing their health-related quality of life (HRQoL). This study examined factors associated with HRQoL among patients with diabetes and CKD.

**Methods:**

A cross sectional study among adults with comorbid diabetes and CKD (eGFR <60 mL/min/1.73m^2^) recruited from renal and diabetes clinics of four large tertiary referral hospitals in Australia was performed. Each participant completed the Kidney Disease Quality of Life (KDQoL ^™^ -36) questionnaire, which is comprised of two composite measures of physical and mental health and 3 kidney disease specific subscales with possible scores ranging from 0 to 100 with higher values indicating better HRQoL. Demographic and clinical data were also collected. Regression analyses were performed to determine the relationship between HRQoL and potential predictor factors.

**Results:**

A total of 308 patients were studied with a mean age of 66.9 (SD = 11.0) years and 70% were males. Mean scores for the physical composite summary, mental composite summary, symptom/problem list, effects of kidney disease and burden of kidney disease scales were 35.2, 47.0, 73.8, 72.5 and 59.8 respectively. Younger age was associated with lower scores in all subscales except for the physical composite summary. Female gender, obese or normal weight rather than overweight, and smoking were all associated with lower scores in one or more subscales. Scores were progressively lower with more advanced stage of CKD (*p*<0.05) in all subscales except for the mental composite summary.

**Conclusion:**

In patients with diabetes and CKD, younger age was associated with lower scores in all HRQoL subscales except the physical composite summary and female gender, obese or normal weight and more advanced stages of CKD were associated with lower scores in one or more subscales. Identifying these factors will inform the timely implementation of interventions to improve the quality of life of these patients.

## Introduction

People are living longer, but with an increased burden of chronic disease [1]. This is partly due to advances in medical treatment of chronic diseases such as diabetes and chronic kidney disease (CKD) [2]. Diabetes is increasing in prevalence with 382 million people worldwide, or 8.3% of adults, estimated to have diabetes and by 2035, some 592 million people, or one adult in 10, will have diabetes [3]. Given that CKD is a common complication of diabetes, the number of patients with diabetes requiring dialysis is also likely to increase. Contributing factors include an ageing population, increase in prevalence of obesity and improved survival rates after cardiovascular events [4].

HRQoL is an indicator of the impact of a condition on a patient’s life and well-being [5]. Patients with diabetes and CKD have significantly impaired HRQoL [6–9] which may worsen as the disease progresses [5]. Lower HRQoL scores are strongly associated with higher risk of death and hospitalisation [2, 4, 10–12] and poorer glycaemic control in patients with diabetes [13]. Assessment of HRQoL allows for identification of factors that may be targeted to improve patient well-being. Effective interventional strategies to enhance HRQoL may then be implemented [5].

Previous studies have assessed HRQoL in people with either diabetes or CKD but not people with diabetes and CKD [14]. As people with these two chronic diseases are known to have competing physical and psychological needs when compared to people with the single condition, there is a need to understand how their complex needs translate into impact on HRQoL and its specific subscales as well as the impact of increasing disease severity. Within this context there is a need for studies across the continuum from early stages of diabetes and CKD through to late stages [8] that seek to identify factors associated with HRQoL particularly those that can be modified [15]. To do this we examined factors associated with HRQoL in patients with co-morbid diabetes and CKD of varying severity who access specialist medical care from tertiary hospitals.

## Methods

### Study design and participants

This was a cross sectional study of patients attending diabetes and renal outpatient clinics of four public and tertiary hospitals in Victoria and New South Wales (Monash Health, Alfred Health, Royal North Shore Hospital and Concord Hospital) between 2013 and December 2014. Participants were eligible if they received their usual care at one of these hospitals, were fluent in English and had a diagnosis of diabetes (either type 1 or type 2) and CKD stages 3 to 5 (eGFR<60 mL/min/1.73 m^2^) including dialysis. As patients with CKD stages 1 to 2 were excluded from the study albuminuria or proteinuria was not used in the staging of CKD. The diagnosis of diabetes followed the World Health Organisation definition [16] and was recorded from patients’ prior inpatient or outpatient contacts. Patients were recruited prospectively from clinics and asked to complete the Kidney Disease Quality of Life short form (KDQoL ^™^ -36) (S1 Appendix). The questionnaire was self-administered. For each patient a clinical survey was also completed by the site study staff or the clinician. Using standardised procedures, information was extracted from the patient’s medical record. The data included demographic and disease-specific characteristics such as gender, age, body mass index, diabetes type, diabetes duration, estimated glomerular filtration rate (eGFR), treatments including dialysis requirement and type, complications/comorbidities, and glycated hemoglobin (HbA1c) (S2 Appendix). All participants provided written informed consent. The study was approved by Monash University and the respective health service human research ethics committees.

### Demographic and clinical variables

Age, gender, socio-economic status, smoking, body mass index (BMI), stage of kidney disease, duration of kidney disease, duration of diabetes, cardiovascular risk factors (hypertension and dislipidemia) and diabetes complications (retinopathy, peripheral vascular disease and nephropathy) were all recorded as possible determinants of HRQoL.

Socio-economic measures were estimated using the Australian Bureau of Statistics data [17]. Postcodes were coded according to the Index of Relative Social Disadvantage (IRSD), a composite measure based on selected census variables which include income, educational attainment and employment status. The IRSD scores for each postcode were then grouped into quintiles for analysis, where the highest quintile comprised 20% of postcodes with the highest IRSD scores (the most advantaged areas).

BMI (kg/m^2^) was calculated by dividing participants’ weight (in kilograms) by the square of their height (in meters). BMI was categorized into four groups which are underweight (≤18.5 kg/m^2^), normal weight (18.5–24.9 kg/m^2^), overweight (25.0–29.9 kg/m^2^) and obese (≥30.0 kg/m^2^) according to the World Health Organization (WHO) classification [18].

CKD stage as defined by the Kidney Disease Outcomes Quality Initiative (KDOQI) was used to define severity of the disease [19]. Duration of CKD was analysed as a continuous variable and also dichotomised by median duration (to less than 5 years or greater or equal to 5 years). eGFR was calculated using the CKD Epi formula GFR = 141 X min (Scr/κ, 1) ^α^ X max (Scr/κ, 1)^-1.209^ X 0.993^Age^ X 1.018 X 1.159 where Scr is serum creatinine (mg/dL), κ is 0.7 for females and 0.9 for males, α is –0.329 for females and –0.411 for males, min indicates the minimum of Scr/κ or 1, and max indicates the maximum of Scr/κ or 1 [20].

### Outcomes

Health related quality of life was assessed using the English version of the Kidney Disease and Quality of Life (KDQoL^™^-36) questionnaire. This is a 36-item HRQoL survey with five subscales namely the SF-12 measure of physical and mental functioning, burden of kidney disease, symptom/problems list and the effects of kidney disease subscales [21]. Item scores were summed for each scale and transformed on a scale of 0 to 100 with a higher score indicating better HRQoL [21]. The scores of the two summary measures and the total SF-36 are based on the average of the respective scale components.

### Statistical analysis

To determine the factors associated with HRQoL, crude and adjusted analyses of the 5 HRQoL subscales were performed using univariate and multiple linear regression methods. The HRQoL subscales were considered as dependent variables and the socio-demographic and clinical variables were considered as independent variables. Variables included in the multivariable model had a significance level of *p*<0.10. Parameter estimates were examined by backward elimination after every iteration to derive a parsimonious model. Differences in HRQoL across stages of kidney disease were assessed by the chi-squared test for linear trend. Finally, subgroup analysis by dialysis status as well as gender, were tested using two-sample t-test or ANOVA for continuous variables. Results were considered significant at conventional *p*<0.05 level. All analyses were performed with IBM SPSS version 22 (Armonk, NY: IBM Corp.) or Stata version 12.1 (Statacorp, College Station, TX). All *p* values were calculated using two-tailed tests.

## Results

### Patient characteristics

**Fig 1:**
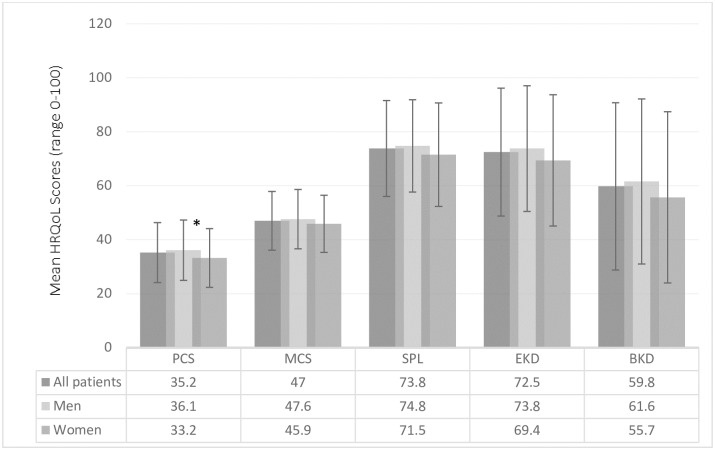
Mean scores for the physical composite summary (PCS), mental composite summary (MCS), symptom/problem list (SPL), effects of kidney disease (EKD) and burden of kidney disease (BKD) subscales. *- Scores significantly different between men and women (*p*<0.05)

Of the 317 patients who participated in the study, 9 were excluded from the analysis due to misclassification of the severity of their kidney disease (eGFR>60 mL/min/1.73 m^2^). The demographic and clinical characteristics of the study population are shown in Table 1. The mean (±SD) age of the cohort was 66.9 ±11 years and 70% were male. The majority of participants were born in Australia (44%) and 78% spoke English as their first language. The median duration of CKD and diabetes were 5 years and 18 years respectively. The means (±SD) for HbA1c and eGFR were 6.8±2.5% (51 mmol/mol) and 29.1±16.7 mL/min/1.73m^2^ respectively. The mean scores for the physical composite summary, mental composite summary, symptom/problem list, effects of kidney disease and burden of kidney disease scales were 35.2 ± 11, 47.0 ± 10.9, 73.8 ± 17.8, 72.5 ± 23.7 and 59.8 ± 31.0 respectively (Fig 1 and Table 2).

**Table 1:** Demographic and clinical characteristics of participants (n = 308).

Characteristic	Value N (SD/%)
N	308
Age (in years)	66.9±11.0
Male	214 (69.5)
**Ethnicity**	
Australian	136 (44.2)
Sri Lanka	16 (5.2)
Greece	13 (4.2)
Italy	12 (3.9)
England	12 (3.9)
Others	119 (38.6)
**Language**	
English	239 (77.6)
Greek	13 (4.2)
Italian	7 (2.3)
Cantonese	5 (1.6)
Mandarin	5 (1.6)
Others	39 (12.7)
**Clinical characteristics**	
HbA1c	6.8±2.5
eGFR	29.1±16.6
Diabetes duration (years), median (IQR)	18 (17)
CKD duration (years), median (IQR)	5 (8)
Body Mass Index (kg/m^2^), median (IQR)	29.9 (8.3)
Smoking status (Yes)	18 (5.8)
Stages of chronic kidney disease	
3a	72 (22.9)
B	79 (25.8)
4	76 (23.5)
5 (on dialysis)	59 (19.2)
5 (not on dialysis)	22 (7.1)

Data are means ± SD or *n* (%); SD-Standard deviation; %- Percentage; HbA1c-glycated haemoglobin; eGFR-estimated Glomerular Filtration Rate.

**Table 2:** Mean health-related quality of life scores of diabetes and chronic kidney disease patients by dialysis status.

SF-36 subscales	All patients	Dialysis	Not on dialysis	*p*-value
		Mean (SD)	Mean (SD)	
Physical composite	35.2±11.1	33.0 (10.3)	35.7 (11.3)	0.10
Mental composite	47.0±10.9	46.4 (10.1)	47.2 (11.1)	0.61
Symptom/Problem List	73.8±17.8	70.8 (16.0)	74.5 (18.1)	0.15
Effects of Kidney Disease	72.5±23.7	58.1 (22.3)	76.0 (22.7)	0.000*
Burden of Kidney Disease	59.8±31.0	33.8 (24.7)	66.3 (28.9)	0.000*

*p-values were significant at 0.05; SD-standard deviation

### Association between patient characteristics and HRQoL

**Fig 2:**
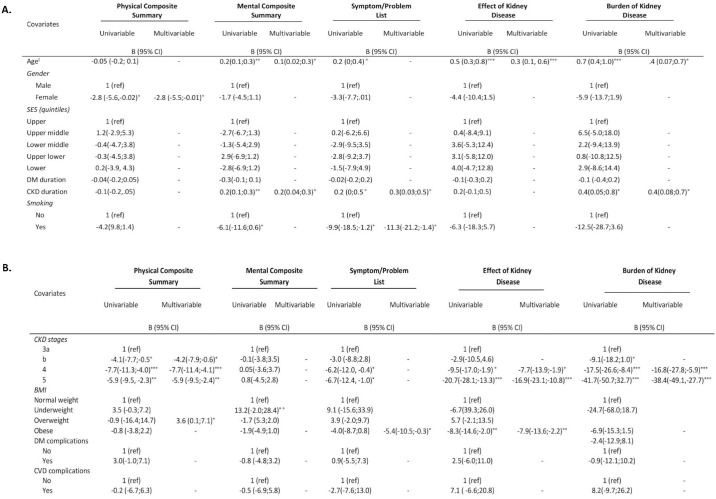
Summary of factors predicting health-related quality of life in patients with diabetes and chronic kidney disease. ĭThe regression coefficient is for a one year increase in age; * p<0.05; **p<0.01; ***p<0.001; SES-Socio Economic Status; DM-Diabetes Mellitus; CKD-Chronic Kidney Disease; CVD-Cardiovascular Disease; BMI-Body Mass Index.

Patient factors associated with HRQoL subscales are shown in Fig 2A and 2B. In multivariable analysis, younger age was associated with lower HRQoL scores in the mental composite summary, effect of kidney disease and burden of kidney disease subscales (all *p* values <0.05). Female patients had lower scores in all subscales compared to male patients but the difference was only significant for the physical composite scale, with female patients scoring on average 3 points lower than their male counterparts (Fig 1). Patients with a BMI in the obese range scored lower on the symptom/problem list and effect of kidney disease subscales than patients with a BMI in the normal range but patients with a BMI in the normal range scored lower on the physical composite summary subscale than patients with a BMI in the overweight range (all *p* values in adjusted analyses <0.05). Smokers scored on average 11 points lower than non-smokers in the symptom/problem list subscale. No associations between socio-economic status and any HRQoL subscales were observed.

### Association between disease severity, duration and HRQoL

**Fig 3:**
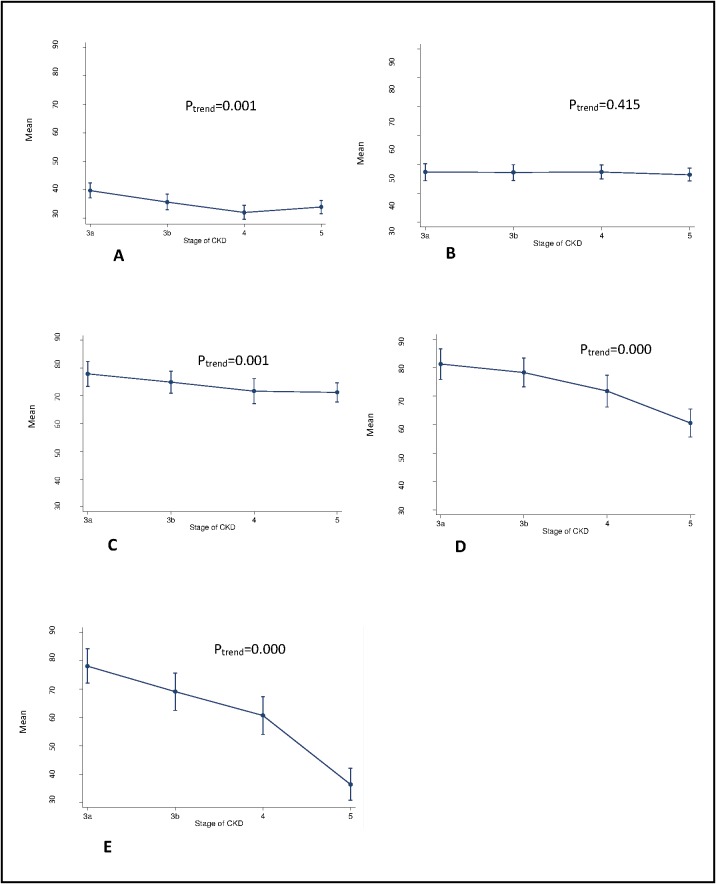
The mean for the health related quality of life subscales. (A) physical composite summary, (B) mental composite summary, (C) symptom problem list, (D) effect of kidney disease and (E) burden of kidney disease scores by stage of kidney disease. Error bars are 95% CI.

With increasing severity of CKD, the mean HRQoL subscales scores decreased significantly except for the mental composite summary subscale (Fig 3). For the physical composite summary, effects of kidney disease, and burden of kidney disease subscales patients in stages 3b, 4 and 5 scored 4–38 points lower than patients in stage 3a (reference group). When the HRQoL scores of dialysis and non-dialysis patients were compared, all the HRQoL subscale scores were lower for dialysis patients but only significantly so for the effect of kidney disease and burden of kidney disease subscales (Table 2).

A shorter duration of CKD was associated with lower scores for mental composite summary, symptom/problem list and burden of kidney disease subscale (all *p* values for adjusted analyses <0.05) (Fig 2A). There was no interaction between the effects of duration of CKD and CKD stages on HRQoL (*p* for interaction >0.05). No associations between diabetes duration or diabetes and cardiovascular complications and any HRQoL subscales were observed (Fig 2B).

## Discussion

The data presented here from a large sample of people with diabetes and CKD attending outpatient specialist diabetes and renal clinics report impaired HRQoL in this population. Younger age was associated with lower scores in the mental composite summary, effect of kidney disease and burden of kidney disease subscales. Female gender was associated with lower physical composite summary scores and BMI in the normal or obese range (compared with overweight range) was associated with lower physical composite summary and lower symptom/problem list and effect of kidney disease scores respectively. In addition, more advanced stages of CKD (stage 3b to 5) were associated with lower physical composite summary, effect of kidney disease and burden of kidney disease scores whilst a shorter duration of CKD was associated with lower mental composite summary, symptom/problem list and burden of kidney disease scores.

Our findings build upon previous research in patients with diabetes [22] or CKD [6] by providing a detailed exploration of factors independently associated with HRQoL across each of the HRQoL subscales and CKD stages in patients with both diabetes and CKD.

We show that people with diabetes and CKD have lower scores in physical and mental composite summary subscales compared to the general population [6]. The mean scores for the HRQoL subscales were comparable to findings from previous studies in patients with kidney disease alone [23, 24] (see S1 Fig) except for the effect of kidney disease and burden of kidney disease subscales where our participants appeared to have scored higher than participants in the ADEMEX [23] and DOPPS [24] studies. This may be explained by the fact that the majority of patients in our study did not have end stage kidney disease compared to patients in the ADEMEX and DOPPS studies who were receiving renal replacement therapy but who did not have diabetes. Despite expecting the physical composite, mental composite and symptom problem list scores to be less impaired in our population with less advanced CKD and diabetes, these subscales were impaired to a similar level to those of patients on dialysis without diabetes. This suggests that the addition of diabetes adds to the burden of disease impacting HRQoL thus physical composite, mental composite and symptom problem list scores to a similar extent as that of dialysis.

Patients with diabetes and more advanced CKD had significantly lower HRQoL mean scores across physical composite summary, symptoms of kidney disease, effect of kidney disease, and burden of kidney disease scores compared to less advanced CKD. It is of interest to note that the decline in HRQoL is apparent well before dialysis has commenced and increases with progression of disease suggesting the need for support for patients at earlier as well as later stages of CKD. In addition, shorter rather than longer duration of CKD was associated with lower mental composite summary, symptom/problem list and burden of kidney disease scores. One explanation may be that as patients become accustomed to their disease over time, they cope better mentally.

In contrast to widely held views and previous reports in patients with CKD alone, [25–27] our data suggest a positive association between age and HRQoL subscales in those with diabetes and CKD. A possible explanation for this relationship, especially for the mental composite summary subscale is that older patients may have better emotional well-being [6]. We speculate that the reason younger patients had lower scores in the burden of kidney disease subscale is that they experience a larger gap between their expected and actual HRQoL and may therefore score lower on HRQoL assessments than older patients, whose experiences are more aligned with their expectations as highlighted by one previous study [28]. This highlights the need to consider and address HRQoL issues in younger people with CKD.

In our study, women scored lower in all HRQoL subscales, but significantly so only for the physical composite summary. Although data for the diabetes and CKD population has not been previously reported, our results are consistent with those of previous studies of populations with advanced CKD [5, 12, 29–31] and may be explained by the fact that women appear to suffer more from chronic illnesses as has also been suggested by studies of populations with vascular diseases [32–34]. Another explanation could be that women, who are also more likely to be care givers than men, may suffer from additional care giver stress [35]. Our failure to demonstrate an association between gender and other HRQoL subscales could be attributed to the smaller number of women surveyed (only 30% of our study population were female).

Patients with a BMI in the normal range scored lower on the physical composite summary subscale than patients with a BMI in the overweight range (BMI of 25–29.9 kg/m^2^). A possible explanation is that being overweight is now more common such that normal weight in patients with diabetes and CKD may reflect more severe disease or the presence of other illnesses. Although seemingly counter-intuitive, wide-ranging benefits of overweight but not obese status among patients with CKD have previously been reported [36, 37]. In contrast, others [5] have reported significantly lower physical composite summary scores among patients who were overweight in CKD stages 2–5, with a GFR ranging from 69 down to 2 ml/min/1.73 m^2^. Of note, patients with a BMI in the obese range also scored lower for symptom/problem list and effect of kidney disease subscales. Further research seeking to determine the ideal BMI for improved survival and HRQoL in this patient population is needed if the reverse epidemiology of being overweight in patients with CKD and diabetes is to be understood.

Our findings should be interpreted in light of design strengths and limitations. The strengths include the inclusion of several biologic and non-biological patient factors as potential predictors for HRQoL in the study population since the likely factors are multifactorial and an even distribution of patients across each KDOQI stage of CKD [19]. We also used a valid and reliable tool (KDQoL^™^-36) for measuring HRQoL. The limitations include a skewed gender distribution with a majority of participants being males, but this is consistent with previously reported gender distribution of studies of patients with CKD [38]. The cross sectional design of the study did not permit us to assess temporal effects. Longitudinal studies need to be conducted to seek a better understanding of factors associated with HRQoL in patients with diabetes and CKD. Additionally, because only participants speaking English were included, our findings cannot be generalised to culturally and linguistically diverse (CALD) populations who may benefit from targeted interventions.

In patients with diabetes and CKD, younger age was associated with lower scores in all HRQoL subscales except the physical composite summary. Additionally, female gender, obese or normal weight, shorter duration of CKD and increasing severity of CKD were associated with lower HRQoL scores in various HRQoL subscales. Identification of these factors informs interventions to improve the quality of life of these patients.

## Supporting Information

S1 Fig Mean HRQoL scores for the physical composite summary (PCS), mental composite summary (MCS), symptom/problem list (SPL), effects of kidney disease (EKD) and burden of kidney disease (BKD) in the ADEMEX study [23], the Dialysis Outcomes and Practice Patterns Study (DOPPS) divided by region [24] and the present study (*Diabetes Renal Project). (TIF)

S1 Appendix Kidney Disease and Quality of Life (KDQOL^™^-36) questionnaire. Measures participants’ health related quality of life.
(PDF)

S2 Appendix Diabetes Renal Project questionnaire. Asks questions about participants’ health indicators.
(PDF)
