# Supplementary figures and images for: Predictors of Health-Related Quality of Life in Patients with Co-Morbid Diabetes and Chronic Kidney Disease
Source: PLoS One. 2016 Dec 19;11(12):e0168491. doi: 10.1371/journal.pone.0168491 (PMC5167387; doi:10.1371/journal.pone.0168491)

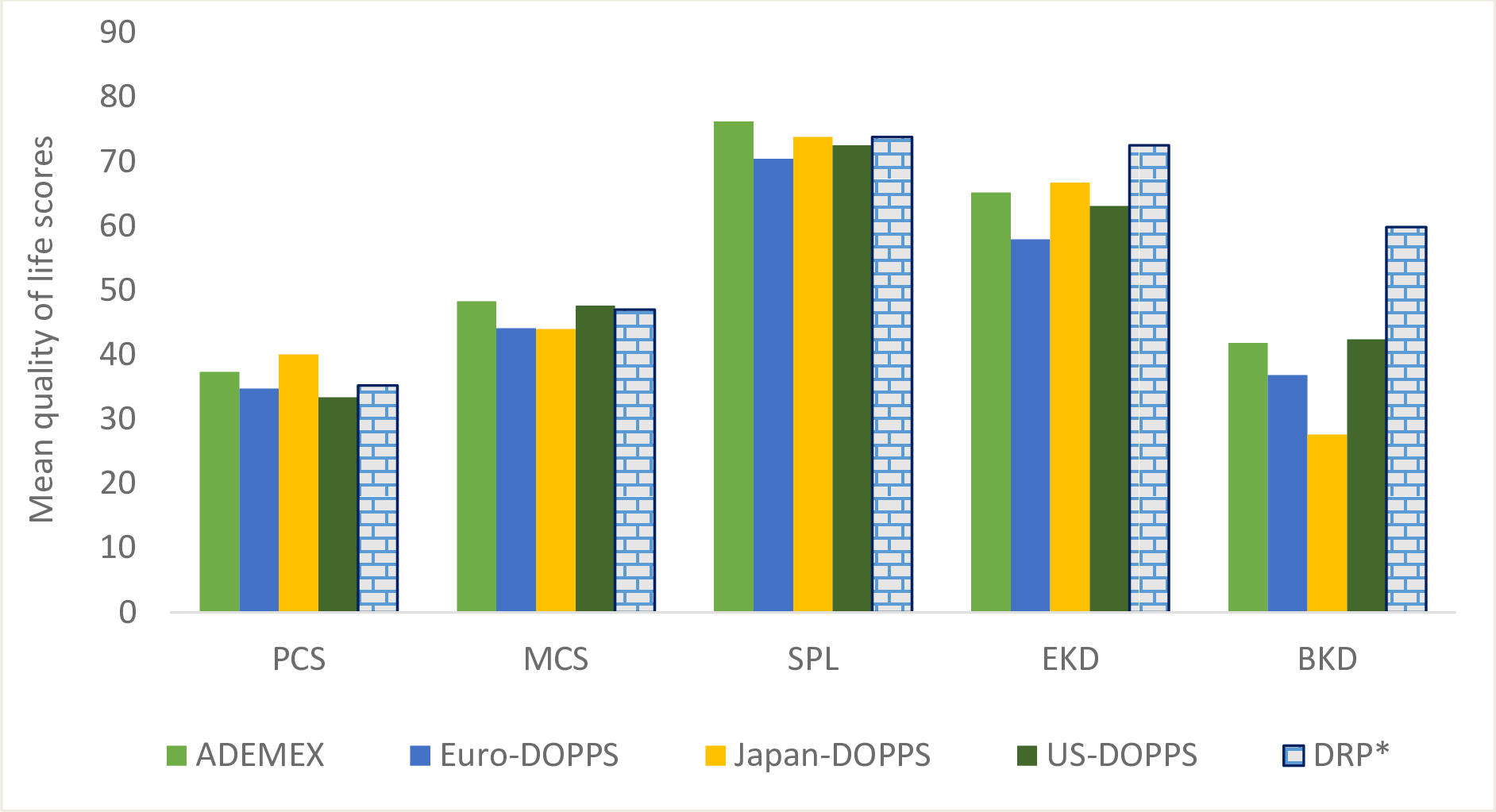

Supplement: S1 Fig — (TIF) [file pone.0168491.s001.tif]
